# Radiological evidence of rapid growth acceleration of a small part solid nodule found to be large-cell carcinoma of the lung

**DOI:** 10.1186/s13019-023-02290-6

**Published:** 2023-07-24

**Authors:** Yunwei Lu, Zewen Sun, Hao Li, Xiaofeng Chen, Yun Li, Lina Sun, Fan Yang, Guanchao Jiang, Jian Zhou

**Affiliations:** Department of Thoracic Surgery, Peking University People’s Hospital, No.11 Xizhimen South Street, Beijing, 100044 China

**Keywords:** Large-cell neuroendocrine carcinoma, Ground-glass nodule, Volume doubling time, Next-generation sequencing, EML4-ALK gene fusion

## Abstract

**Background:**

With the widespread use of low-dose computed tomography for lung cancer screening, the detection rate of pulmonary lesions manifesting as ground-glass opacities (GGOs) has been increasing dramatically. The volume doubling time (VDT) has been introduced in clinical practice to monitor the potential growth rate of GGOs during long-term follow-up periods.

**Case presentation:**

A 72-year-old never-smoker female diagnosed with mixed GGO manifested abruptly accelerated growth with sudden decreased VDT from 400 to 36 days. A thoracoscopic left lower lobectomy with mediastinal lymph node dissection was performed, and the diagnosis was stage IB large-cell neuroendocrine carcinoma (LCNEC). Next-generation sequencing of the tumor highlights an EML4-ALK gene fusion.

**Conclusions:**

The LCNEC may present as GGO with longer VDT in the early stage. VDT should calculate by the whole size either on the entire tumor diameter or on consolidation diameter. It is recommended that meticulous long-term follow-up with dynamic VDT monitoring may help select high-risk GGOs performing timely semi-elective surgical resection in clinical practice.

## Background

The ground-glass opacities (GGOs) are a highly heterogeneous disease. Generally, they are considered early-stage primary lung cancer with indolent tumor behavior and excellent prognosis. However, a small proportion of GGOs may harbor aggressive growth patterns with early intrapulmonary metastasis. Therefore, the volume doubling time (VDT) has provided a monitor for the potential growth rate of GGOs during long-term follow-up periods. In this case, an abruptly decreased VDT from 400 to merely 36 days might reflect the tumor’s greater histological aggressiveness, which correlated well with its neuroendocrine origin.

## Case presentation

**Fig. 1:**
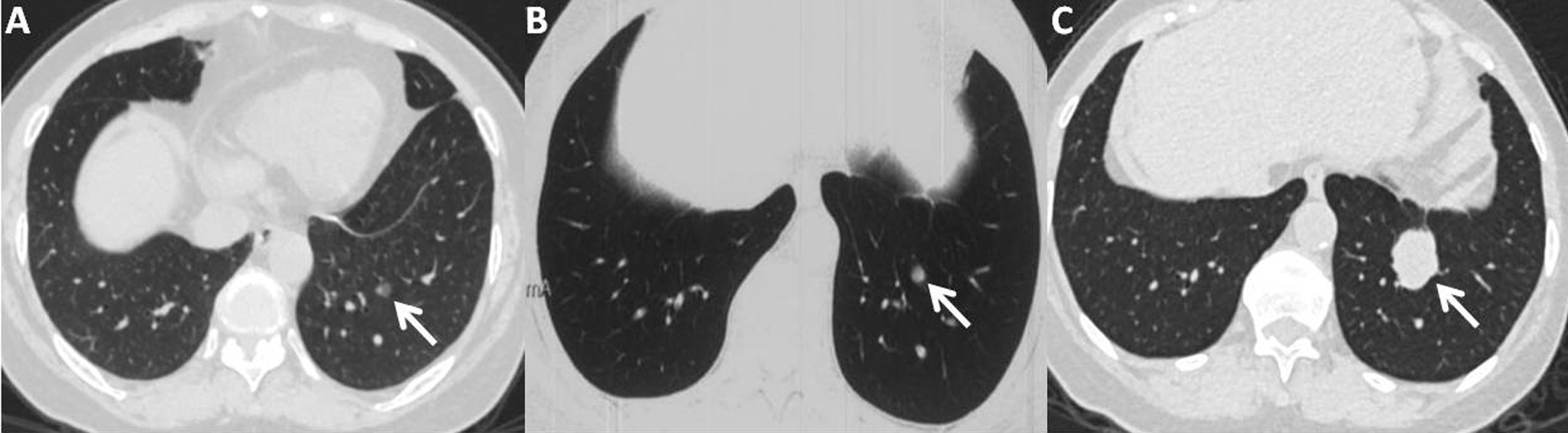
**A** The chest CT on 29th December, 2019 revealed a 0.8 cm × 0.6 cm mixed GGO lesion with a distinct boundary on her left lower lobe, which comprised 3 mm subsolid components; **B** The second chest CT on 15th September, 2020 showed that there was no significant change in the size of this nodule, but revealed a larger solid component within the mixed GGO lesion; **C** The third chest CT on 19th April, 2021 showed a 3.1 cm × 2.9 cm completely solid nodule with discriminated boundary and sign of lobulation

This patient is a 72-year-old never-smoker female in good general condition with no chief complaints. She has no significant past medical history of neoplastic disease or any risk factor associated with lung disease, except for bilateral carotid plaques. From a lung cancer screening in December 2019, the initial chest CT scan incidentally detected a mixed GGO at the left lower lung (Fig. 1A). The total size of the nodule was 0.8 cm × 0.6 cm, while the maximum diameter of the solid component was 0.3 cm. A second chest CT scan was performed to confirm the nodule’s persistence in September 2020 (Fig. 1B). Although the dimension of the nodule remained essentially unchanged, the attenuation of the mixed GGO increased remarkably during this 9-month follow-up interval. No surgical intervention was performed at this time since there was no direct evidence regarding cancer probability in such a small nodule with low clinical risk. Due to the small size of the lesion, no experimental treatment with antibiotics was implemented for this patient. However, a more intense follow-up schedule was recommended.

In April 2021, a third chest CT scan conducted seven months after the second showed that the mixed GGO had already progressed to a 3.1 cm × 2.9 cm solid tumor with an obvious lobulation sign (Fig. 1C). VDTs calculated by the Modified Schwartz Equation were 400 and 36 days respectively throughout the two follow-up periods. The Fluorodeoxyglucose positron emission tomography (FDG-PET) scan exhibited a high maximum standardized uptake value (SUVmax: 5.1) lesion at the left lower lobe. This patient detected no distant metastasis by PET/CT or contrast-enhanced brain magnetic resonance imaging (MRI). Serum tumor biomarker levels, such as carcinoembryonic antigen (CEA: 6.19ng/ml) and progastrin-releasing peptide (ProGRP: 81.8pg/ml), were elevated in this case.

**Fig. 2:**
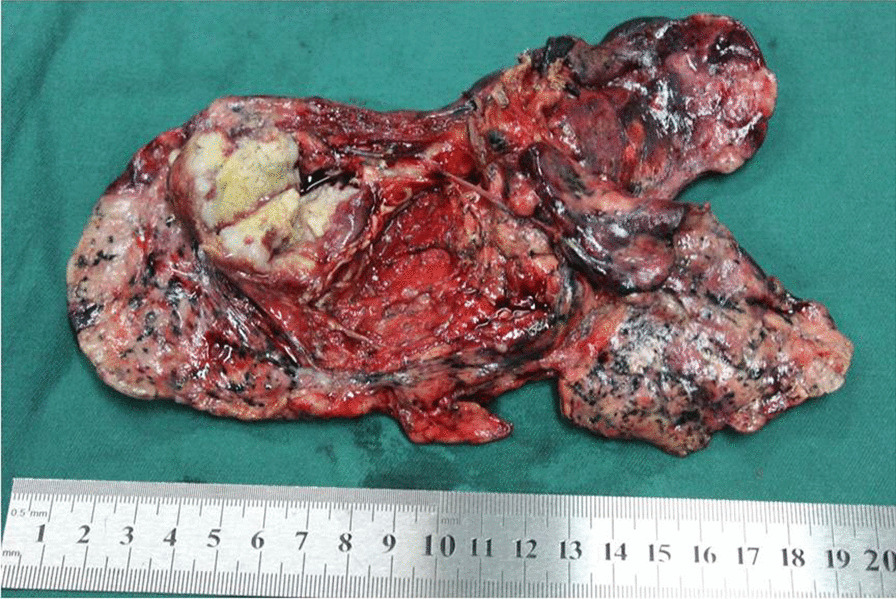
The left lower lobectomy specimen disclosed a firm, yellow-tan mass 3.2 cm × 3.1 cm in size, and surgical margins appeared to be negative and wide from the tumor

**Fig. 3:**
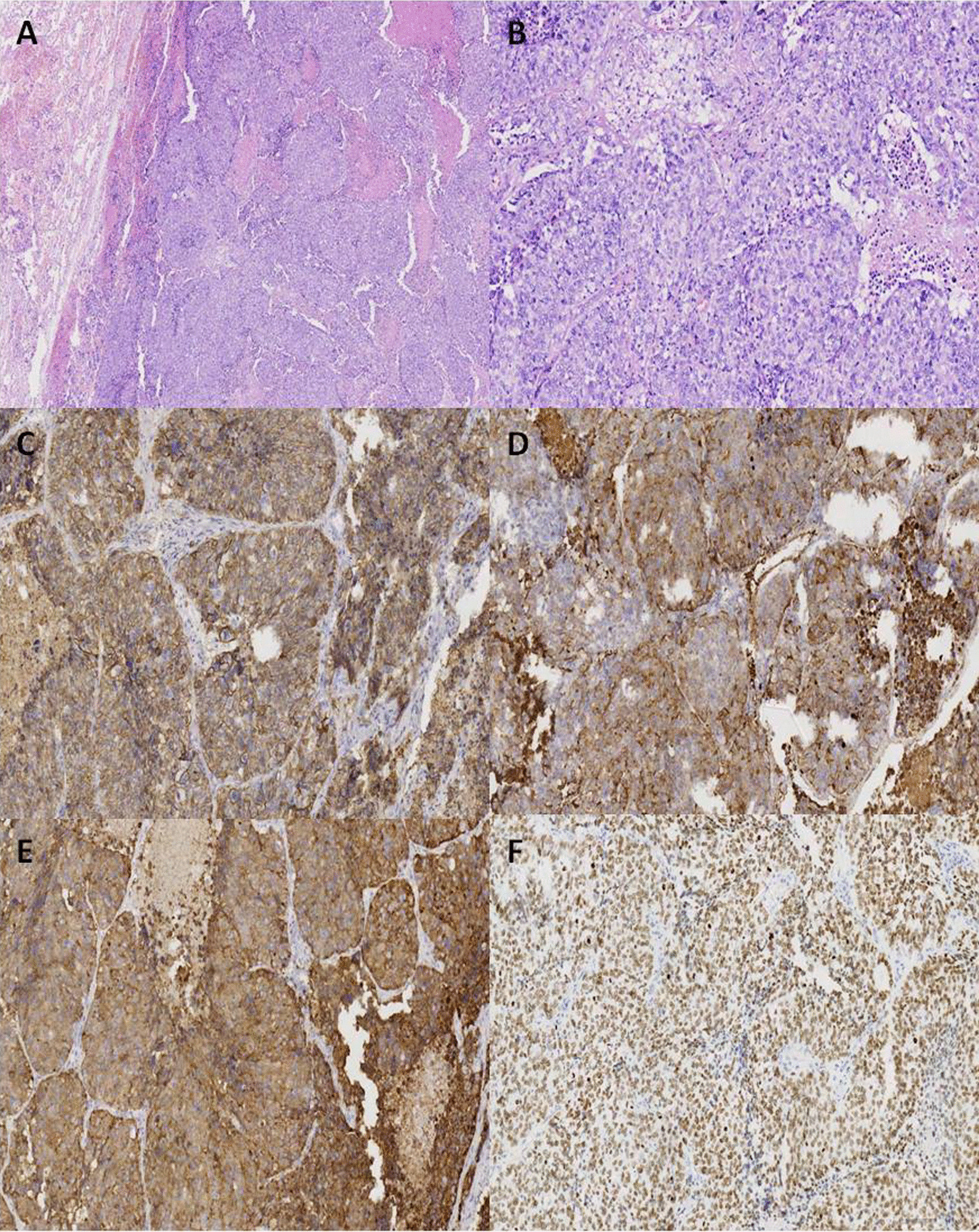
**A** 5× magnification. **B**, **C**, **D**, **E**, **F** 20× magnification. **A**, **B** The tumor cells present pleomorphic and large with abundant cytoplasm, large and vesicular nucleoli with hematoxylin and eosin (HE) staining; **C** negative with CD5/6 staining; **D** positive with CgA staining; **E** positive with Syn staining; **F** positive with TTF-1 staining

The patient underwent thoracoscopic left lower lobectomy with mediastinal lymph node dissection and was diagnosed with stage IB (pT2aN0M0, the 8th AJCC edition) LCNEC. Surgical specimens were examined both grossly (Fig. 2) and microscopically after Hematoxylin and Eosin (H&E, Fig. 3A and B) and immunohistochemistry staining (IHC, Fig. 3C, F). A 654-gene broad-panel NGS spanning 1.60 megabases (Mb) of the human genome was then performed (Berry Oncology, Beijing, China). Five single nucleotide variations (SNVs: *TP53*, *RB1*, *MUC16*, *LATS2*, *STAG2*), three copy number amplifications (CNVs: *CSF3R*, *MPL*, *TERT*), and one gene fusion (*EML4*-*ALK*) were identified in this sample. The patient’s postoperative course was unremarkable. She was discharged on postoperative day 8. Platinum-based adjuvant chemotherapy was recommended for the patient. The patient showed no signs of postoperative complications or recurrence 16 months after surgery.

## Discussion and conclusions

VDT is defined as the number of days in which a nodule doubles its volume [1]. It plays an essential role in differentiating benign and malignant pulmonary nodules, thus determining the follow-up strategy and the timing of surgical intervention. Generally, a shorter VDT indicates rapid growth, aggressive tumor behavior, and poor cancer prognosis. According to the results derived from the NELSON study, a VDT of less than 400 days should be considered the best cut-off value to distinguish between indolent and malignant pulmonary nodules [2]. Moreover, for solid nodules, VDTs are well established, with a large majority of times being in the 100-400-day range. While for subsolid nodules, longer VDTs are expected following their more indolent tumor behavior. Hasegawa et al. reported that the mean VDTs for pure GGOs, mixed GGOs, and solid nodules were 813, 457, and 149 days, respectively [3]. For this reason, longer initial follow-up intervals and more extended total follow-up periods are recommended for subsolid nodules than for solid ones [4].

Although pulmonary lesions manifesting as GGOs are typically early-stage lepidic-predominant adenocarcinoma with indolent tumor behavior and excellent prognosis, few of them may harbor aggressive histological type and have a rapid tumor growth rate. As is well known, the tumor growth rate is highly variable and relates to the histological type. Mackintosh et al. demonstrated that the median VDTs for lung adenocarcinoma, squamous cell carcinoma, and LCNEC were 261, 70, and 45 days, respectively [5]. A shorter VDT represents a poorly differentiated tumor. In this case, an abruptly decreased VDT from 400 to merely 36 days might reflect the tumor’s greater histological aggressiveness, which correlated well with its neuroendocrine origin. Therefore, GGOs with long VDT do not guarantee an indolent tumor growth pattern. Meticulous long-term follow-up with dynamic VDT monitoring may help select high-risk GGOs requiring timely semi-elective surgical resection in clinical practice. Initial large size, suspicious morphology, a growing solid component, the presence of bubble lucency, and a history of cancer were generally accepted risk factors for the growth and malignant transformation of GGOs [6]. For these very suspicious GGOs, a short-term follow-up CT scan at 3–6 months was recommended in the guidelines [7].

LCNECs are rare high-grade lung neuroendocrine tumors with poor prognosis. Genetically, LCNECs possess a high mutation rate (8.5–10.5 mutations/Mb), similar to small cell lung cancer (SCLC). Recently, Derks et al. [8] dichotomized two major molecular subtypes of LCNECs: one with the inactivation of *TP53* and *RB1*, a hallmark of SCLC (SCLC-type); the other with the inactivation of *TP53* and *STK*11/*KEAP1*/*RAS* pathway genes, which are frequently mutated in non-small cell lung cancer (NSCLC-type). Preliminary data showed that those as mentioned above two molecular subtypes might have predictive value for chemotherapy response. Compared with the traditional NSCLC chemotherapy regimen (gemcitabine/pemetrexed/paclitaxel combined with platinum), SCLC-type tumor, like in this case, may have a better outcome when treated with platinum-etoposide [9]. Besides, our NGS data highlighted an EML4-ALK gene fusion in this LCNEC. EML4-ALK is most often detected in never smokers. It is associated with early tumor metastasis due to its potently oncogenic effect [10].

In summary, chest CT provides a vital opportunity for timely detecting pulmonary lesions. Thus, an increasing number of GGOs were identified, in which VDT plays an essential role in the therapeutic method. It is recommended that long-term follow-up with dynamic VDT monitoring and perform timely semi-elective surgical resection in clinical practice. Furthermore, NGS may assist clinicians in detecting gene mutation, further understanding the character of the tumor, and guiding targeted therapy.

## Data Availability

All data are available in the manuscript within the manuscript.

## Abbreviations

CEA: Carcinoembryonic antigen
CT: Computed tomography
FDG-PET: Fluorodeoxyglucose positron emission tomography
GGO: Ground-glassopacity
H&E: Hematoxylin and Eosin
IHC: Immunohistochemistry staining
LCNEC: Large-cell neuroendocrine carcinoma
Mb: Megabases
MRI: Magnetic resonance imaging
NGS: Next-generation sequencing
ProGRP: Progastrin-releasing peptide
SCLC: Small cell lung cancer
SNV: Single nucleotide variations
VDT: Volume doubling time
